# Factors influencing pharmacy program choice and career attitudes among Tanzanian tertiary students

**DOI:** 10.1186/s12909-026-09431-0

**Published:** 2026-05-21

**Authors:** Calvin Andrea, Justine Methusela, Auson Magige, Fredon Teingisa, Caroline Meena, Mariki Urasa, David Mwaipaya, Evance Mgeyi

**Affiliations:** 1 St John’s University of Tanzania, P.O. Box 47, Dodoma, Tanzania; 2 Pharmacy Council of Tanzania, P.O. Box 1277, Dodoma, Tanzania; 3 Famasi360, P.O Box 1135, Dodoma, Tanzania; 4 Muhimbili National Hospital - Mloganzila, P.O. Box 65000, Dar es Salaam, Tanzania; 5 Africa Health Network, P. O. Box 23037, Dar es Salaam, Tanzania; 6 Baylor College of Medicine Children’s Foundation, Tanzania, P.O. Box 5208, Mwanza, Tanzania

**Keywords:** Pharmacy education, Career choice, Professional attitudes, Tanzania, Pharmacy students, Cross-sectional study

## Abstract

**Background:**

While pharmacy education has expanded in Tanzania, students’ motivations for choosing the profession and their career expectations remain underexplored. Understanding these drivers is crucial for aligning training with healthcare needs, enhancing professional satisfaction, and ensuring graduates contribute effectively to national health priorities.

**Methods:**

A descriptive cross-sectional study was conducted between March and July 2025 among 324 undergraduate pharmacy students conveniently sampled from St. John’s University of Tanzania (SJUT), DECCA College of Health and Allied Science (DECOHAS), and Kampala International University of Tanzania (KIUT) in Tanzania. Data were collected using a structured questionnaire and analysed using STATA version 17. Descriptive statistics, chi-square tests, and multivariate logistic regression were employed to identify associations between factors influencing program choice and career attitudes, with statistical significance set at *p* < 0.05.

**Results:**

Pharmacy was the first-choice program for 76.2% of participants, and 53.7% demonstrated positive career attitudes (*p* = 0.01). Significant associations with selecting pharmacy as first choice were found for institution attended (χ²=13.587, *p* = 0.001), program level (χ²=13.862, *p* < 0.001), confidence in choice (χ²=7.438, *p* = 0.006), program reputation (χ²=14.75, *p* < 0.001), source of influence (χ²=14.742, *p* = 0.002), advice from pharmacists/healthcare workers (χ²=4.105, *p* = 0.043), and attitudinal disposition (χ²=7.342, *p* = 0.007). After adjustment, significant predictors of choosing pharmacy first included low confidence (AOR = 0.33, *p* = 0.046), program reputation (AOR = 0.39, *p* = 0.017), negative attitude (AOR = 0.54, *p* = 0.047), and community pharmacy career goals (AOR = 3.82, *p* = 0.045).

**Conclusion:**

Pharmacy program choice among Tanzanian students is influenced by institutional factors, professional reputation, mentorship from healthcare professionals, and attitudinal disposition rather than financial incentives. Structured mentorship programs and enhanced career guidance are recommended to align student expectations with workforce needs.

## Background

Over the past decades, pharmacy has shifted from a product-focused discipline centred on compounding and dispensing medicines to a patient-centred clinical profession embedded within multidisciplinary healthcare teams [1–3]. This transition has been driven by increasing health system complexity, the rising burden of chronic diseases, and the global need for safe and rational medicine use [4, 5], leading to expanded pharmacist roles in medication therapy management, counselling, adherence support, and clinical decision-making [6]. Despite this expanded scope and global recognition by WHO and FIP, substantial workforce disparities remain, with recommended pharmacist-to-population ratios of about 1: 2,000 rarely achieved in low- and middle-income countries, where ratios may reach 1: 10,000–1: 20,000 due to training limitations, migration, and weak retention systems [1, 7]. In Tanzania, these shortages are more pronounced, particularly in rural settings, constraining effective integration of pharmacists into clinical care despite ongoing health system strengthening efforts [7, 8].

Pharmacy is widely perceived as a respected and stable profession, and students’ career choices are influenced by a mix of intrinsic and extrinsic factors, including interest in science and medicine, desire to help others, job security, financial prospects, professional status, and external pressures such as family expectations and societal prestige [2, 9–14].

Evidence shows that students motivated by genuine interest and commitment to healthcare are more likely to experience professional fulfilment and pursue advanced clinical or academic roles, whereas those driven mainly by social or economic pressures may report lower satisfaction or leave the profession [15–19].

In Tanzania, this global challenge is reflected in a rapidly evolving educational landscape [4, 8, 13]. The expansion of Bachelor of Pharmacy (BPharm) programs over the past two decades has increased student enrolment and diversity [4, 8, 13]. However, a critical disconnect has emerged between student aspirations and national health needs [4, 8, 13]. Evidence suggests that many pharmacy graduates in Tanzania favour private-sector and urban employment, contributing to a workforce misdistribution that leaves critical gaps in public and rural health facilities where the need for pharmaceutical care is most acute [4, 8, 13]. This trend threatens the equitable distribution of the pharmaceutical workforce and the country’s ability to achieve its health goals [4, 8, 13]. Understanding the motivations and attitudes of students at entry into the pharmacy profession is therefore essential for informing targeted educational, mentorship, and policy interventions that can better align training outputs with national workforce needs and correct existing maldistribution patterns. This study aimed to determine the factors influencing students’ choice of pharmacy as a field of study and to assess their attitudes toward future careers within the profession.

## Methods

### Study design and setting

A descriptive cross-sectional study was conducted between March and July 2025 among undergraduate pharmacy students enrolled in selected tertiary institutions in Tanzania. The study was carried out in three institutions: St. John’s University of Tanzania (SJUT) and DECCA College of Health and Allied Sciences (DECOHAS) in Dodoma, and Kampala International University in Tanzania (KIUT) in Dar es Salaam. These institutions were selected due to their large and diverse pharmacy student populations, representing different training levels and institutional contexts.

### Study population and eligibility criteria

The target population included all undergraduate students enrolled in Bachelor’s or Diploma in Pharmacy programmes at selected institutions (*N* = 2,300). Eligible participants were aged ≥ 18 years, present during data collection, and provided informed consent. Students not meeting these criteria or unwilling to participate were excluded (*n* = 600), resulting in a final study population of 1,700 participants.

### Sample size and sampling procedure

The sample size (*n* = 324) was calculated using a finite population correction, assuming a population of 1,700 eligible students, a 95% confidence level (Z = 1.96), a proportion of 0.5, and a 5% margin of error. Participants were recruited using convenience sampling, with all eligible students present during data collection invited until the target sample was reached. This approach may limit sample representativeness.

### Data collection tools and procedures

Data were collected using a self-administered structured questionnaire assessing pharmacy students’ career preferences, influencing factors, and professional attitudes. Validity was ensured through review by two senior experts and pilot testing among 30 selected students. Feedback informed instrument adjustment, and pilot data were excluded from analysis. Internal consistency was acceptable (Cronbach’s α = 0.80). The questionnaire comprised three sections: socio-demographic characteristics (age, gender, institution, year of study); factors influencing the choice of pharmacy (e.g., personal interest, family influence, academic background, job security, financial motivation); and attitude toward the pharmacy profession, measured using multiple items aggregated into a composite score. Attitude scores were standardized using Z-scores and classified using a cut-off of 0, with < 0 indicating negative and ≥ 0 indicating positive attitudinal disposition.

### Data management and statistical analysis

Data were entered, cleaned, and analysed using STATA version 17. Descriptive statistics summarized participant characteristics as frequencies, percentages, means, and standard deviations. Bivariate analysis using Pearson’s chi-square (χ²) test assessed associations between independent variables and the outcome (selection of pharmacy as a first-choice program). Variables with *p* < 0.05 in bivariate analysis, along with key covariates (age, sex, institution, program level, and year of study), were included in a multivariable logistic regression model to control for potential confounding. Adjusted odds ratios (AORs) with 95% confidence intervals (CIs) were reported. Statistical significance was set at *p* < 0.05. No substantial missing data were observed.

## Results

### Socio-demographic characteristics

A total of 324 students participated in the study, yielding a response rate of 100%. The mean age of participants was 23.1 ± 3.2 years, with ages ranging from 17 to 36 years (95% CI [22.7–23.4], IQR = 4). The sample was nearly gender-balanced with higher representation among female students (166, 51.2%). The largest proportion of respondents, 143 (44.1%), were from SJUT, followed by 133 (41.1%) from DECOHAS, and 48 (14.8%) from KIUT.

In terms of academic program, 163 (50.3%) students were enrolled in the degree program, while 161 (49.7%) were pursuing a diploma in pharmacy. The distribution by year of study showed that 33.6% were in Year 1, 21.3% in Year 2, 24.7% in Year 3, and 20.4% in Year 4. This distribution reflects a balanced representation across academic levels and provides insight into perceptions formed during different stages of training.

Regarding their program preference, 247 (76.2%) students indicated that pharmacy was their first choice of study at the time of admission. The remaining 77 (23.8%) students reported that pharmacy had been their second choice.

### Attitude toward a career in pharmacy

The mean attitude score among respondents was 6.60 ± 1.33 (SD = 1.33; 95% CI: 6.46–6.75), with a median of 7.00 (IQR = 7). Attitude scores were standardized using z-scores and categorized as positive (z ≥ 0) or negative (z < 0). Overall, 174 (53.7%) students demonstrated a positive attitude, while 150 (46.3%) demonstrated a negative attitude (*p* = 0.01).

### Factors associated with selecting pharmacy as a first-choice program

Table 1 presents the results of chi-square tests examining associations between socio-demographic and motivational variables and the likelihood of selecting pharmacy as a first-choice program. Participant age, sex, and year of study were not significantly associated with selecting pharmacy as a first. This suggests that demographic characteristics alone did not meaningfully influence the decision to choose the pharmacy program as a first-choice program.

**Table 1:** Association between participant characteristics and the selection of pharmacy as the first-choice program (Bivariate analysis with Chi-square test)

Category	Pharmacy as First Choice				
	Yes (*n*, Adj. Res)	No (*n*, Adj. Res)	*n* (%)	χ²	*p*-value
Age Group				3.62	0.31
Below 19	31 (1.8)	4 (-1.8)	35 (10.8%)		
20–24	144 (-0.8)	49 (0.8)	193 (59.6%)		
25–29	63 (-0.5)	22 (0.5)	85 (26.2%)		
Above 29	9 (0.4)	2 (-0.4)	11 (3.4%)		
Sex				2.84	0.092
Male	114 (-1.7)	44 (1.7)	158 (48.8%)		
Female	133 (1.7)	33 (-1.7)	166 (51.2%)		
University / College				13.59	0.001*
SJUT	95 (-3.7)	48 (3.7)	143 (44.1%)		
KIUT	40 (1.3)	8 (-1.3)	48 (14.8%)		
DECOHAS	112 (2.8)	21 (-2.8)	133 (41.0%)		
Program of Study				13.86	< 0.001*
Degree	110 (-3.7)	53 (3.7)	163 (50.3%)		
Diploma	137 (3.7)	24 (-3.7)	161 (49.7%)		
Year of Study				4.51	0.211
First Year	90 (1.9)	19 (-1.9)	109 (33.6%)		
Second Year	50 (-0.8)	19 (0.8)	69 (21.3%)		
Third Year	61 (0.0)	19 (0.0)	80 (24.7%)		
Fourth Year	46 (-1.4)	20 (1.4)	66 (20.4%)		
Personal Interest in Pharmacy				1.20	0.273
Low	27 (-1.1)	12 (1.1)	39 (12.0%)		
High	220 (1.1)	65 (-1.1)	285 (88.0%)		
Confidence in Choice				7.44	0.006*
Low	25 (-2.7)	17 (2.7)	42 (13.0%)		
High	222 (2.7)	60 (-2.7)	282 (87.0%)		
Secondary School Performance Influence				0.31	0.579
No	35 (0.6)	9 (-0.6)	44 (13.6%)		
Yes	212 (-0.6)	68 (0.6)	280 (86.4%)		
Main Source of Influence				14.74	0.002*
Parents / Family	174 (-0.8)	58 (0.8)	232 (71.6%)		
Friends / Peers	14 (-2.8)	12 (2.8)	26 (8.0%)		
Teachers / Mentors	41 (2.8)	3 (-2.8)	44 (13.6%)		
Social media	18 (0.6)	4 (-0.6)	22 (6.8%)		
Pharmacy Program Reputation				14.75	< 0.001*
No	35 (-3.8)	26 (3.8)	61 (18.8%)		
Yes	212 (3.8)	51 (-3.8)	263 (81.2%)		
Financial Factors (Future Earnings)				0.62	0.431
No	81 (-0.8)	29 (0.8)	110 (34.0%)		
Yes	166 (0.8)	48 (-0.8)	214 (66.0%)		
Job Market Opportunities				0.97	0.324
No	75 (-1.0)	28 (1.0)	103 (31.8%)		
Yes	172 (1.0)	49 (-1.0)	221 (68.2%)		
Advised by Healthcare Worker				4.11	0.043*
No	78 (-2.0)	34 (2.0)	112 (34.6%)		
Yes	169 (2.0)	43 (-2.0)	212 (65.4%)		
Family Member in Healthcare Advice				0.01	0.937
No	79 (-0.1)	25 (0.1)	104 (32.1%)		
Yes	168 (0.1)	52 (-0.1)	220 (67.9%)		
Long-Term Career Goals				0.85	0.838
Community Pharmacy	74 (0.7)	20 (-0.7)	94 (29.0%)		
Hospital Pharmacy	90 (0.2)	27 (-0.2)	117 (36.1%)		
Pharmaceutical Industry	59 (-0.6)	21 (0.6)	80 (24.7%)		
Academic / Research	24 (-0.5)	9 (0.5)	33 (10.2%)		
Attitudinal Disposition				7.34	0.007*
Negative	104 (-2.7)	46 (2.7)	150 (46.3%)		
Positive	143 (2.7)	31 (-2.7)	174 (53.7%)		

*Adj. Res* Adjusted residuals examined to identify cells contributing to significant associations; absolute values greater than 2 (|Adj. Res| > 2) indicate that the observed frequency differs significantly from the expected frequency, *DECOHAS* Decca College of Health and Allied Science, *HCW* Healthcare Worker, *KIUT* Kampala International University in Tanzania, *n* Sample size, *SJUT* St. John’s University of Tanzania, *χ*² Chi-square

*Statistically significant are bolded

University/college attended and program of study demonstrated strong and statistically significant associations with pharmacy being a first choice (University: χ²=13.59, *p* = 0.001; Program: χ²=13.86, *p* < 0.001). Students enrolled at DECOHAS showed a higher likelihood of selecting pharmacy as their first choice (Adj. Res = 2.8), whereas those from SJUT were less likely (Adj. Res = − 3.7). Similarly, Diploma students were significantly more inclined to choose pharmacy first (Adj. Res = 3.7), compared to Degree students (Adj. Res = − 3.7).

Confidence in choice showed a significant association (χ²=7.44, *p* = 0.01) with their choice in pharmacy, where those with high confidence selected pharmacy as their first choice [*n* = 222 (87.0%), Adj. Res = 2.7], compared to those with low confidence were less represented (*n* = 42 (23%), Adj. Res = − 2.7).

The main source of influence was statistically significantly associated with pharmacy choice (χ²=14.74, *p* = 0.002). Guidance from teachers/mentors and social media was positively associated with choosing pharmacy first (Adj. Res = 2.8, Adj. Res = 0.6, respectively), whereas influence from friends/peers was negatively associated (Adj. Res = − 2.8). Additionally, being advised by a pharmacist or healthcare worker was also significant (χ²=4.11, *p* = 0.04), with those receiving such advice being more likely to select pharmacy first (Adj. Res = 2.0).

The reputation of the pharmacy program was associated with the student choosing pharmacy (χ²=14.75, *p* < 0.001). Those who acknowledged program reputation as influential were more likely to choose pharmacy as their first choice (Adj. Res = 3.8), indicating the strong effect and belief towards the Pharmacy profession and training institutions. Finally, attitudinal disposition toward pharmacy was also significant (χ²=7.342, *p* = 0.007); respondents with a positive attitude were more likely to select pharmacy first (Adj. Res = 2.7), while those with a negative disposition were less likely (Adj. Res = − 2.7.

### Predictors of choosing pharmacy as the first choice

Table 2 shows that, in the bivariate analysis, several factors were significantly associated with the choice of pharmacy as a career. Students from St. John’s University of Tanzania had significantly lower odds of choosing pharmacy compared to those from DECOHAS (cOR = 0.37, 95% CI: 0.21–0.66, p = 0.001*). Being enrolled in a degree program was also associated with lower odds of selecting pharmacy (cOR = 0.36, 95% CI: 0.21–0.63, *p* < 0.001). First-year students were more likely to choose pharmacy compared to fourth-year students (cOR = 2.06, 95% CI: 1.00–4.24, *p* = 0.05). Low confidence in career choice (cOR = 0.40, 95% CI: 0.20–0.78, *p* = 0.008), influence from friends/peers compared to social media (cOR = 0.26, 95% CI: 0.07–0.98, *p* = 0.05), lack of program reputation influence (cOR = 0.32, 95% CI: 0.18–0.59, *p* < 0.001), not being advised by a pharmacist/HCW (cOR = 0.58, 95% CI: 0.35–0.99, *p* = 0.044), and negative attitudinal disposition (cOR = 0.49, 95% CI: 0.29–0.83, *p* = 0.007) were all significantly associated with lower odds of choosing pharmacy.

**Table 2:** Bivariate and Multivariate Logistic Regression Analysis of Factors Associated with Choice of Pharmacy Career among University Students

Influencing Factors	Reference Category	cOR (95% CI)	*p*-value	AOR (95% CI)	*p*-value
Grouped Participant Age^a^					
Below 19	Above 29	1.72 (0.27–10.98)	0.57	0.67 (0.07–7.00)	0.74
20–24	Above 29	0.65 (0.14–3.13)	0.59	0.41 (0.06–2.85)	0.37
25–29	Above 29	0.64 (0.13–3.18)	0.58	0.72 (0.11–4.86)	0.74
Sex^a^					
Male	Female	0.64 (0.38–1.08)	0.09	0.95 (0.50–1.79)	0.87
University or College^a^					
SJUT	DECOHAS	**0.37 (0.21–0.66)**	0.001*	2.01 (0.53–7.62)	0.30
KIUT	DECOHAS	0.94 (0.39–2.29)	0.89	9.06 (1.82–45.11)	0.007*
Program of study^a^					
Degree	Diploma	**0.36 (0.21–0.63)**	< 0.001*	0.13 (0.03–0.48)	0.002*
Year of Study^a^					
First Year	Fourth Year	**2.06 (1.00–4.24)**	0.05*	1.02 (0.36–2.94)	0.97
Second Year	Fourth Year	1.14 (0.54–2.41)	0.72	0.57 (0.21–1.56)	0.27
Third Year	Fourth Year	1.40 (0.67–2.91)	0.37	0.95 (0.38–2.37)	0.91
Personal Interest in Pharmacy					
Low	High	0.67 (0.32–1.39)	0.28	1.45 (0.45–4.64)	0.53
Confidence in Choice					
Low	High	**0.40 (0.20–0.78)**	0.008*	0.33 (0.11–0.98)	0.046*
Secondary School Performance Influence					
No	Yes	1.25 (0.57–2.73)	0.58	1.58 (0.62–4.04)	0.34
Main Source of Influence					
Parents / Family	Social Media / Online	0.67 (0.22–2.05)	0.48	0.41 (0.11–1.48)	0.17
Friends / Peers	Social Media / Online	**0.26 (0.07–0.98)**	0.047*	0.23 (0.05–1.04)	0.06
Teachers / Mentors	Social Media / Online	3.04 (0.62–14.99)	0.17	2.65 (0.47–15.11)	0.27
Program Reputation Influence					
No	Yes	**0.32 (0.18–0.59)**	< 0.001*	0.39 (0.18–0.84)	0.017*
Future Financial Factors Influence					
No	Yes	0.81 (0.47–1.38)	0.43	1.35 (0.68–2.68)	0.39
Job Market Influence					
No	Yes	0.76 (0.45–1.31)	0.32	1.20 (0.60–2.38)	0.61
Advised by Pharmacist / HCW					
No	Yes	**0.58 (0.35–0.99)**	0.044*	0.70 (0.36–1.34)	0.28
Advised by Family in Healthcare/Pharmacy					
No	Yes	0.98 (0.57–1.69)	0.94	1.07 (0.54–2.14)	0.85
Long-Term Career Goals after Graduation					
Community Pharmacy	Academic / Research	1.39 (0.56–3.45)	0.48	3.82 (1.033–14.118)	**0.045**
Hospital Pharmacy	Academic / Research	1.25 (0.52–3.01)	0.62	2.02 (0.62–6.55)	0.24
Pharmaceutical Industry	Academic / Research	1.05 (0.42–2.63)	0.91	1.78 (0.58–5.52)	0.32
Participants Attitudinal Disposition					
Negative	Positive	0.49 (0.29–0.83)	0.007*	0.54 (0.29–0.99)	0.047*
Constant				24.13	0.018*

*AOR* Adjusted Odds Ratio, *CI* Confidence Interval, *cOR *Crude Odds Ration, *df* Degrees of Freedom, *HCW*  Healthcare Worker, *SJUT* St. John’s University of Tanzania, *KIUT* Kampala International University in Tanzania

*Significant are bolded. Variables marked^a^ (age, sex, university/college, program of study, and year of study) were included in the multivariable model as adjustment covariates; their estimates are presented for control of confounding rather than primary interpretation. *p* < 0.05 indicates statistical significance

A multivariate logistic regression model was developed using the enter method. Multicollinearity among predictors was assessed, with tolerance values > 0.2 and variance inflation factors (VIF) < 5, indicating no significant collinearity. Model fit was evaluated using the Hosmer–Lemeshow test, which showed an adequate fit (χ²=4.864, df = 8, *p* = 0.772).

After adjusting for age, sex, institution, program of study, and year of study, only a subset of variables remained independently significant. Students with low confidence in their career choice had 67% lower odds of selecting pharmacy (AOR = 0.33, 95% CI: 0.11–0.98, *p* = 0.046). Those who reported no influence from program reputation were significantly less likely to choose pharmacy (AOR = 0.39, 95% CI: 0.18–0.84, *p* = 0.017). Students aspiring to practice in community pharmacy were nearly four times more likely to choose pharmacy compared to those intending to pursue academic or research careers (AOR = 3.82, 95% CI: 1.03–14.12, *p* = 0.045). Additionally, a negative attitudinal disposition remained significantly associated with reduced odds of choosing pharmacy (AOR = 0.54, 95% CI: 0.29–0.99, *p* = 0.047).

## Discussion

This study examined factors influencing students’ choice of pharmacy as a field of study and their career attitudes, highlighting the interaction between socio-demographic characteristics, motivational drivers, institutional context, and professional perceptions among Tanzanian pharmacy students.

Although gender was not a significant predictor, its balanced distribution reflects a positive shift toward gender equality in pharmacy education, a field historically dominated by men in many African settings. This aligns with global trends where pharmacy has become increasingly attractive to both genders, particularly in high-income countries such as the United Kingdom and the United States, where women now constitute the majority of practicing pharmacists [1, 20] This shift may be attributed to improved access to higher education for women, gender equity initiatives in Science, Technology, Engineering, and Mathematics (STEM) fields, and the perception of pharmacy as a stable and socially respected profession.

Professional reputation emerged as a strong determinant of selecting pharmacy as a first-choice program. Students perceived pharmacy as a prestigious discipline integrating science, healthcare, and community service. This aligns with evidence identifying professional status, social recognition, and altruistic motivation as key drivers of career choice [12]. A comparable pattern was documented in Ethiopia, where perceived social prestige and professional recognition were among the strongest motivators for pharmacy enrolment, particularly among students from urban settings [21]. In Tanzania, this perception may also reflect the expanding clinical role of pharmacists within health systems [8], reinforcing pharmacy’s visibility and attractiveness to prospective students.

Although financial incentives were reported by most participants as influential, they were not independently significant in multivariable analysis. This suggests that financial considerations may represent baseline expectations rather than decisive determinants of career choice. Similar findings have been reported in Sudan and China, where financial motivation loses significance after adjusting for intrinsic and contextual factors [9, 22]. Corroborating evidence from Vietnam similarly showed that although salary was acknowledged, professional reputation and career prospects ranked as stronger independent motivators for pharmacy enrolment [23]. In Tanzania, relatively standardized public-sector remuneration may further limit its differentiating effect across career options.

Family influence was identified as the dominant external factor shaping career decisions. This reflects sociocultural norms in Tanzania and many African contexts, where educational pathways are often guided collectively by families. While parental involvement can reinforce motivation when aligned with student interests, it may also constrain autonomy when driven by social prestige or external pressure. This contrasts with more individualistic decision-making models observed in Western settings [23, 24] but is consistent with findings from other low- and middle-income countries [25]. These findings underscore the need for inclusive career guidance strategies that engage both students and parents.

Exposure to pharmacists and healthcare professionals through mentorship was positively associated with choosing pharmacy as a first-choice program. Such exposure provides realistic insights into professional roles and strengthens career motivation. A study across 25 pharmacy colleges in Saudi Arabia found that internship training influenced career choice, with summer training linked to community pharmacy and clinical rotations to clinical specialization [26]. Similar findings from Sudan and Nigeria demonstrate that early professional interaction enhances interest in pharmacy careers [9]. This highlights the importance of structured mentorship and outreach programs to support informed career decision-making.

Institutional affiliation and program level were also significant predictors. Diploma students were more likely to select pharmacy as their first choice compared to degree students, possibly due to more focused entry pathways and limited alternative options. In contrast, degree students often have broader academic choices and may consider pharmacy as a secondary option following other health-related disciplines [27]. This is supported by South Korean evidence showing stronger motivation and clearer career alignment among students in direct integrated pharmacy programmes compared to pre-pharmacy transfer students [28]. Institutional differences in reputation and academic positioning may further influence student motivation and expectations.

Career aspirations strongly shaped pharmacy choice, with students intending to pursue community pharmacy being 3.8 more likely to select pharmacy as their first choice. Community pharmacy, as the most visible and accessible sector of the profession, provides a clear and relatable career pathway for students [9, 19]. International evidence suggests that students motivated by patient-centred goals tend to favour community and clinical roles, while interest in academia and research remains comparatively low [29]. Some evidence also suggests that a proportion of students enter pharmacy as a secondary choice after not gaining admission into medicine or related fields, which may influence later career orientation [30, 31]. This has implications for professional identity formation and curriculum design.

Finally, students with a positive attitudinal disposition and higher confidence in their career choice were significantly more likely to select pharmacy as their first-choice program. This reflects strong professional commitment and optimism about the future of the profession. Similar patterns have been reported in studies from the United States, Saudi Arabia, Nigeria, Pakistan, and Southeast Asia, where confidence and positive perception are key determinants of sustained engagement in pharmacy careers [15, 17, 19]. Such confidence is essential for long-term motivation, academic persistence, and professional development.

### Bias and study limitations

Several measures were taken to minimize bias, including standardized data collection procedures and the use of a pre-tested questionnaire to reduce information bias. However, the cross-sectional design limits causal inference and does not capture changes over time in students’ motivations or perceptions. The study was conducted in selected institutions, which may limit generalizability. In addition, convenience sampling may have introduced selection bias, affecting representativeness. Finally, the use of a self-reported attitudinal scale may be subject to response bias and may not fully capture the complexity of students’ attitudes.

## Conclusion

This study shows that Tanzanian pharmacy students are mainly influenced by the institution of study, program level, long-term career alignment, sources of influence, the profession’s reputation, professional advice, and personal attitudes, rather than financial incentives. Hospital and community pharmacy were the most common career aspirations, while research and academia were less frequently reported.

### Recommendation

This study recommends strengthening mentorship programs led by pharmaceutical professionals in secondary schools to inspire and guide students toward pharmacy careers. In addition, pharmacy training programs should incorporate structured rural clinical placements and community pharmacy rotations to expose students to underserved settings. Policymakers and training institutions should also consider targeted incentives such as scholarships, bonded placements, and career advancement opportunities in the public sector to encourage pharmacy graduates to work in rural and public healthcare facilities, thereby helping address the existing workforce maldistribution.

## Data Availability

The datasets generated and analyzed during the current study are available from the corresponding author upon reasonable request. Data are not publicly available due to privacy and ethical restrictions, as they contain information that could compromise participant confidentiality.
